# Global and Targeted Pathway Impact of Gliomas on White Matter Integrity Based on Lobar Localization

**DOI:** 10.7759/cureus.1660

**Published:** 2017-09-07

**Authors:** David R Ormond, Shawn D'Souza, John A Thompson

**Affiliations:** 1 Neurosurgery, University of Colorado Hospital; 2 Neuroscience Program, University of Colorado Boulder

**Keywords:** tractography, primary brain glioma, diffusion tensor imaging, intracranial tumor, white matter tracts

## Abstract

Primary brain tumors comprise 28% of all tumors and 80% of malignant tumors. Pathophysiology of high-grade gliomas includes significant distortion of white matter architecture, necrosis, the breakdown of the blood brain barrier, and increased intracranial pressure. Diffusion tensor imaging (DTI), a diffusion weighted imaging technique, can be used to assess white matter architecture. Use of DTI as a non-invasive pathophysiological tool to analyze glioma impact on white matter microstructure has yet to be fully explored.

Preliminary assessment of DTI tractography was done as a measure of intracranial tumor impact on white matter architecture. Specifically, we addressed three questions: 1) whether glioma differentially affects local white matter structure compared to metastasis, 2) whether glioma affects tract integrity of major white matter bundles, 3) whether glioma lobe localization affects tract integrity of different white matter bundles.

In this study, we retrospectively investigated preoperative DTI scans from 24 patients undergoing tumor resection. Fiber tractography was estimated using a deterministic fiber tracking algorithm in DSI (diffusion spectrum imaging) Studio. The automatic anatomical labeling (AAL) atlas was used to define the left and right (L/R)* *hemisphere regions of interest (ROI). In addition, the John Hopkins University (JHU) White Matter Atlas was used to auto-segment major white matter bundle ROIs. For all tracts derived from ROI seed targets, we computed the following parameters: tract number, tract length, fractional anisotropy (FA), axial diffusivity (AD), mean diffusivity (MD), and radial diffusivity (RD).

The DTI tractography analysis revealed that white matter integrity in the hemisphere ipsilateral to intracranial tumor was significantly compromised compared to the control contralateral hemisphere. No differences were observed between high vs low-grade gliomas, however, gliomas induced significantly greater white matter degradation than metastases. In addition, targeted analysis of major white matter bundles important for sensory/motor function (i.e., corticospinal tract and superior longitudinal fasciculus) revealed tract-parameter specific susceptibility due to the presence of the tumor. Finally, major tract bundles were differentially affected based on lobar localization of the glioma.

These DTI-based tractographic analyses complement findings from gross histopathological examination of glioma impact on neural tissue. Global and focal white matter architecture, ipsilateral to glioma, shows higher rates of degradation or edema – based on DTI tractographic metrics – in comparison to normal brain or metastases. Gliomas, which arise in the parietal lobe, also have a higher negative impact (potentially due to increased edema) on white matter integrity of the superior longitudinal fasciculus(SLF) than those which arise in the frontal lobe. Future studies will focus on using preoperative and postoperative tractography to predict functional deficits following resective surgery.

## Introduction

Gliomas are tumors which arise from glial cells, specifically astrocytes, oligodendrocytes, and/or ependymal cells [1]. Gliomas comprise 28% of all tumors and 80% of primary intracranial tumors [2]. Depending on the type, location, and rate of expansion, gliomas can cause damage to surrounding tissue through necrosis and hypoxia [3]. Previous methods of studying glioma impact on the neural milieu were largely used in vitro biochemical or histopathological measures [2]. Several aberrant signaling pathways have been identified that give rise to surrounding tissue damage [4].

However, the impact of a glioma on white matter architecture and integrity is poorly understood. Diffusion tensor imaging (DTI) has been widely accepted as a preoperative neurosurgical imaging tool to assist with the demarcation of gliomas and aid in the preservation of major white matter tract function during intracranial tumor resection [5]. In addition, in the context of several neurological conditions, DTI has been used as an indirect measure of axonal integrity [6]. Four major measures of tract integrity can be derived from estimation of the diffusion coefficient: fractional anisotropy (FA), which shows the degree of anisotropy in a given diffusion area, mean diffusivity (MD) which measures the diffusion of water molecules within a given voxel, independent of anisotropy, axial diffusivity (AD) which refers to the water diffusion parallel to white matter fibers and is proportional to axonal damage, and radial diffusivity (RD) which refers to the water diffusion perpendicular to white matter fibers and is indirectly proportional to demyelination [7]. Recently, studies have begun to utilize this technology to answer how gliomas affect surrounding white matter microstructure [8].

This study demonstrates the use of DTI as an anatomical and pathophysiological tool to better understand the impact gliomas have on the brain by addressing several objectives: 1) assess differences in track number and diffusivity parameters between tumor and non-tumor hemispheres: separate comparisons assessed differences between grade III/IV or grade II glioma and glioma or metastasis, 2) assess glioma impact on targeted major white matter bundles, 3) evaluate the degree to which lobe localization of the glioma effects tract integrity of targeted major sensory-motor related white matter tracts.

## Technical report

Tumor demographics

All procedures and protocols for this study were reviewed and approved by the Colorado Multi-Institutional Review Board (17-1136). Subjects included in this study were patients undergoing resective surgery, from January to December 2016 at the University of Colorado Hospital, to remove an intracranial tumor classified by histopathology as either glioma or metastasis requiring functional imaging due to localization in or near language or motor cortex. Data were collected retrospectively from patient chart review. Glioma cases (n = 17) included in this study were a heterogenous group of both low and high-grade tumors. Similarly, the metastatic tumor cases (n = 7) included in this study arose from many different secondary sites.

Imaging sequence parameters

Magnetic resonance images (MRI) were collected on a 3.0 T GE Signa scanner. MRI parameters were the following: T1-weighted image - field of view (FOV) 24 cm, TR:9.5; flip angle: 20°. DTI - 1 b-value at 1000, 32 directions, axial FOV 24 cm, slice thickness 2.6 mm, spacing 0, frequency direction R to L, TR 15,500, 50 slices, phase/frequency 128, bandwidth 250, phase acceleration 2.0.

DTI preprocessing

All processing steps were conducted in DSI (diffusion spectrum imaging) Studio [9]. DTI was reconstructed from 32 sampling direction image sets and the b0 image (1000) using the approach implemented in DtiStudio [10], in addition to correcting for eddy currents and EPI distortions. Within DSI Studio, the 'DTI' reconstruction method was used to model the eigenvectors.

Whole brain tractography

DSI studio was used to run Streamline (Euler) fiber tracking algorithm [9] on the preoperative DTI of each intracranial tumor case (n = 24). The FA map was saved for each case and used for along-tract analysis.

Interhemispheric differences

The Automatic Anatomical Label (AAL; [11]) atlas was used to define both left and right hemisphere regions-of-interest (ROI). Fiber tracking was run using one hemisphere as the ROI and the opposing hemisphere as a region of avoidance (ROA) (e.g., Left Hemisphere fiber tracking = Left Hemisphere ROI, Right Hemisphere ROA). Track number, FA, MD, RD, and AD were collected for the fiber tracking of each ROI.

Targeted white matter bundle analysis

The John Hopkins University White Matter (JHU-WM; [12]) atlas was used to define L/R corticospinal tract (CST) and superior longitudinal fasciculus (SLF) white matter tract ROIs. Fiber tracking was run using one side as the ROI and the opposing side AAL hemisphere as the ROA. Track number, FA, MD, RD, and AD were collected for the fiber tracking of each ROI.

Along-tract analysis

For fibers extracted using the JHU-WM ROI seed-based approach for the CST and SLF, we computed the mean tract length across subjects clustering the bundles into either tumor or non-tumor groups. Using the mean tract length, a spline interpolation was applied to the x, y, and z coordinates of the fiber representation. Interpolation was used to resample the tracts and fit them to the standard mean length. Distance points along the length of the resampled tracts were compared across groups using a parametric t-test. To control for multiple comparisons, we computed the false discovery rate (FDR; [13]) of the population of significance tests for each diffusion parameter within each bundle (i.e., CST and SLF). Within each diffusion parameter, the adjusted critical p-value derived from FDR was applied to the population of significance tests to determine significance at each point along the tract [14].

Lobe localization effect on major motor pathways

Glioma cases were categorized under frontal, parietal, temporal, or occipital depending on tumor location. Parameters were set to distinguish between tumors near the frontoparietal border. The MRI slice containing the largest brain area was first measured vertically. A horizontal line was drawn across the midpoint of the vertical measurement. >50% of the mass anterior to this line was classified as frontal and >50% of the mass posterior to this line was classified as parietal. Due to insufficient sample size for temporal and occipital cases, these gliomas were excluded from statistical analysis.

Statistics

Appropriate parametric and nonparametric group-wise comparisons were used to assess differences between glioma and non-glioma impact on targeted ROIs. The along-tract analysis employed multiple pairwise comparisons that were corrected by using the false discovery rate adjusted critical value to determine significance.

Glioma impact on white matter integrity

**Figure 1:**
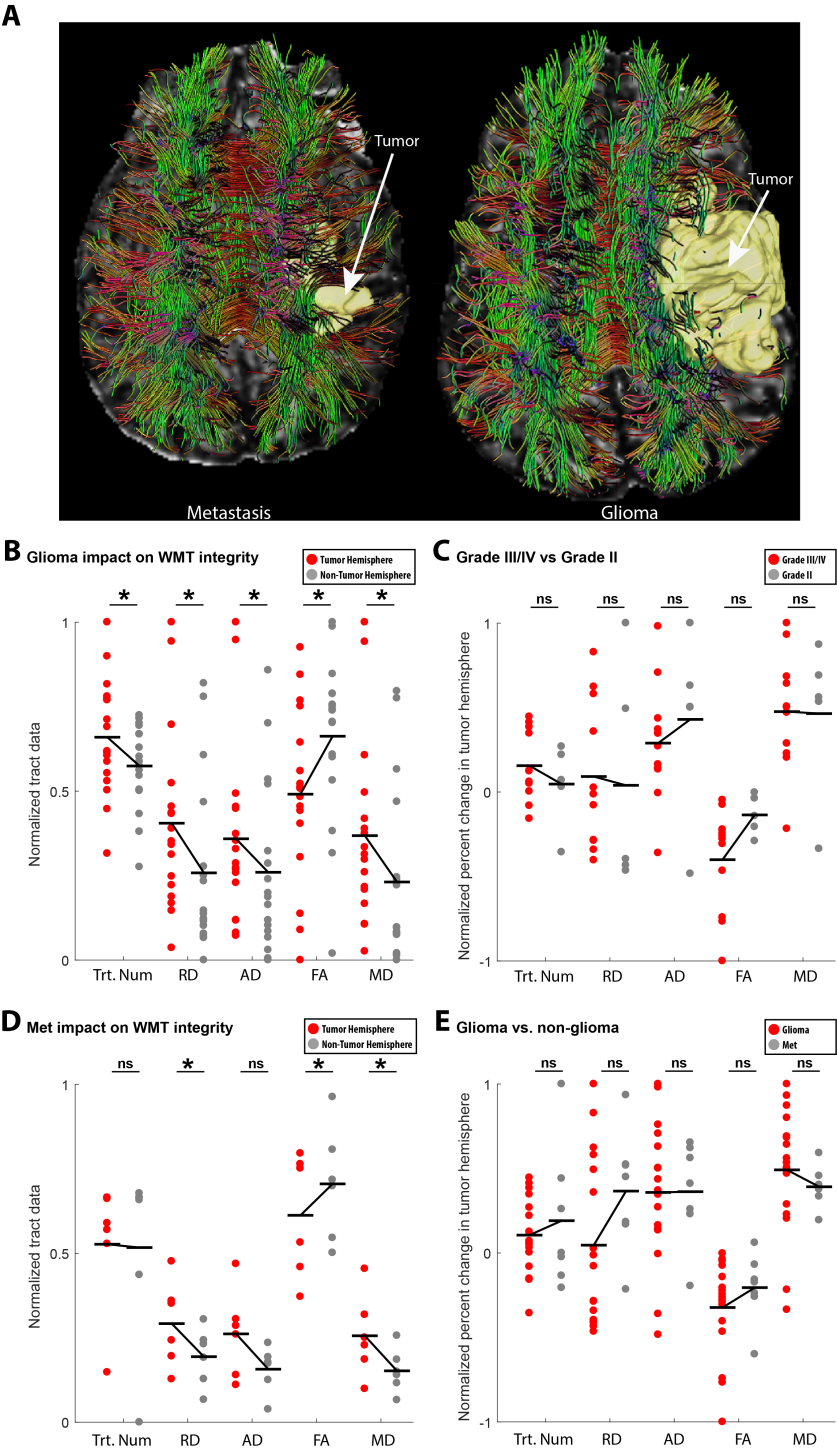
Glioma impact on white matter integrity. (A) Representative image sets for whole brain tractography, tumor segmentation, and FA map (left, metastasis; right, glioma). (B) Comparison of global (hemisphere) white matter impact due to glioma using diffusivity parameters. Changes consistent with white matter degradation were observed on the hemisphere ipsilateral to glioma in tract number, RD (radial diffusivity), AD (axial diffusivity), FA (fractional anisotropy), and MD (mean diffusivity). (C) Comparison of global (hemisphere) white matter impact due to high vs low grade glioma using diffusivity parameters. No differences were observed. (D) Comparison of global (hemisphere) white matter impact due to metastasis using diffusivity parameters. Changes consistent with white matter degradation were observed on the hemisphere ipsilateral to tumor in RD (radial diffusivity), AD (axial diffusivity), FA (fractional anisotropy), and MD (mean diffusivity), but not tract number. (E) Comparison of global (hemisphere) white matter impact between glioma and metastasis using diffusivity parameters. No differences were observed.

Figure 1A shows representative manually traced 3D segmentations for glioma (right) and metastasis (left) overlaid on the DTI-derived whole brain tractography and FA map. To assess whether the presence of glioma affected global white matter structure, we analyzed the interhemispheric difference in DTI estimated fiber tract parameters by comparing the hemisphere ipsilateral to the tumor with respect to the contralateral non-tumor hemisphere. The raw data for each tract parameter – combined tumor and non-tumor data – was normalized using the following feature scaling equation: $\left ( i \right ) = \frac{x\left ( i \right ) - min\left ( x \right )}{max\left ( x \right ) - min\left ( x \right )}$ where x(*i*) represents the *i*th raw parameter value. For tract number, the glioma affected hemisphere exhibited significantly more DTI derived fiber tracts as compared to the subject-specific non-tumor hemisphere (*p* = 0.05, *t* = 2.02, df = 16). For all tracts, we derived standard estimates of diffusivity: AD, RD, FA and MD. All four diffusivity measures showed significant modification as function of the presence of glioma. RD, AD and MD showed an increase (RD, *p* = 0.0012, *t* = 3.92, df = 16; AD, *p* = 0.02, *t* = 2.43, df = 16; MD, *p* = 0.003, *t* = 3.47, df = 16), whereas FA showed a decrease as a function of the presence of a glioma (FA, *p* = 2.51e-05, *t* = -5.84, df = 16; Figure 1B). Given the diversity of glioma classifications present in our data set, we sought to determine whether different grades of glioma differentially impacted global white matter structure. We compared the Grade II gliomas with the Grade III/IV gliomas, which exhibit aggressive, invasive, and rapid growth. We observed no differences for any tract parameter between the Grade II and the more aggressive Grade III/IV gliomas (tract number, *p* = 0.58, *Z* = 99; RD, *p* = 0.4, *Z* = 101; AD, *p* = 0.26, *Z* = 83; FA, *p* = 0.52, *Z* = 76; MD, *p* = 0.66, *Z* = 89; Figure 1C).

Metastasis impact on white matter integrity

In Figure 1D, we estimated the impact of metastasis on DTI-derived fiber tract parameters. Similar to glioma, there were modifications in the measures of diffusivity: increases in RD and MD, and decreases in FA, as a function of the metastasis (RD, *p* = 0.02, *t* = 3.20, df = 5; FA, *p* = 0.04, *t* = -2.8, df = 5; MD, *p* = 0.03, t = 3.02, df = 5). However, unlike glioma, there was no difference in tract number or AD (tract number, *p* = 0.84, *t* = 0.20, df = 5; AD, *p* = 0.07, *t* = 2.32, df = 5). We further directly compared the difference in white matter compromise between glioma and metastasis and found no differences (tract number, *p* = 0.89, *Z* = 215; RD, *p* = 0.12, *Z* = 188; AD, *p* = 1, *Z* = 202; FA, *p* = 0.28, *Z* = 195; MD, *p* = 0.16, *Z* = 235; Figure 1E).

Glioma impact on major white matter tracts with functional implications for resection

**Figure 2:**
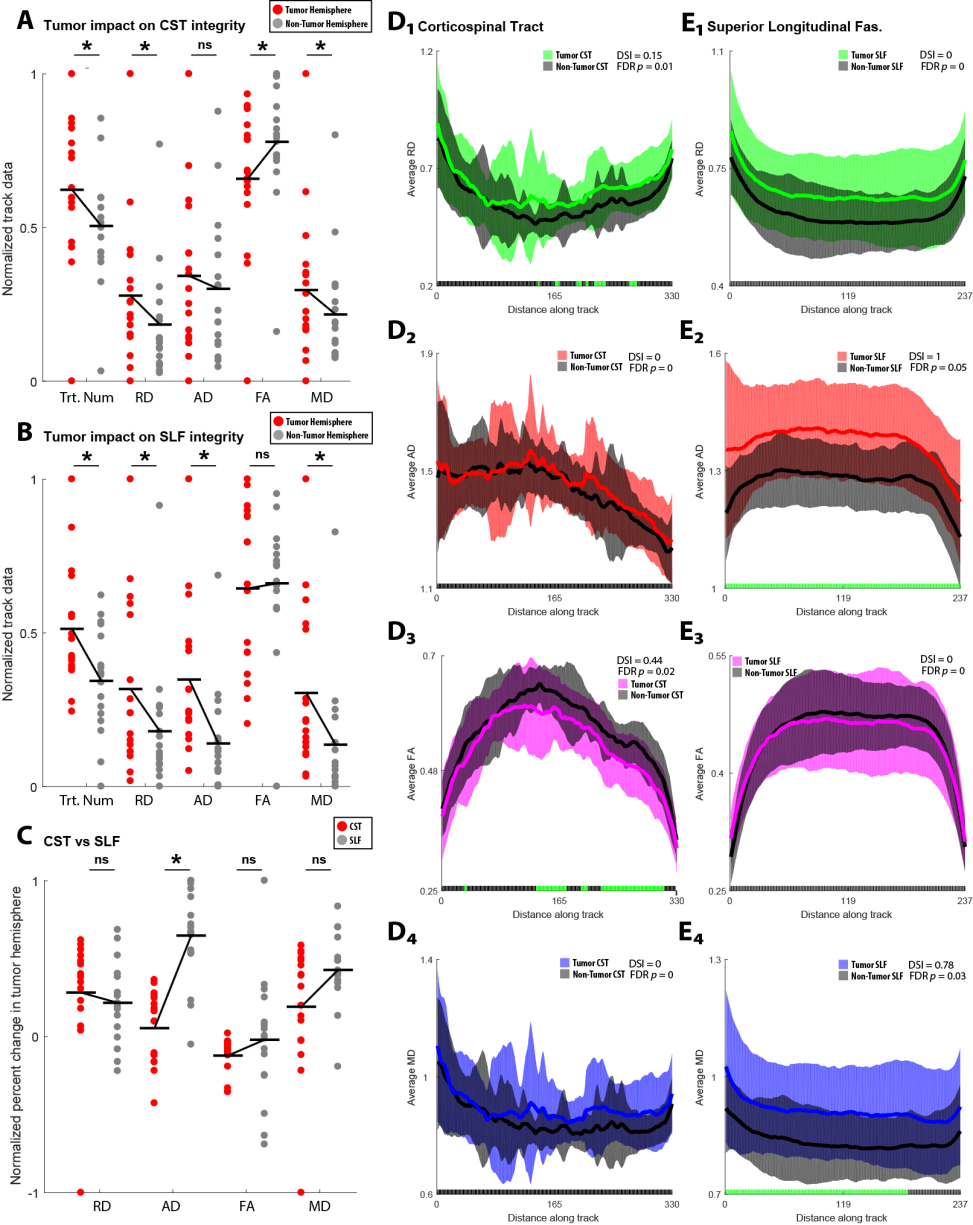
Glioma impact on major white matter tracts with functional implications for resection. (A) Comparison of major tract (corticospinal tract; CST) white matter impact due to glioma using diffusivity parameters. Changes consistent with white matter degradation were observed in the CST ipsilateral to glioma in tract number, RD (radial diffusivity), FA (fractional anisotropy), and MD (mean diffusivity). (B) Comparison of major tract (superior longitudinal fasciculus; SLF) white matter impact due to glioma using diffusivity parameters. Changes consistent with white matter degradation were observed in the SLF ipsilateral to glioma in tract number, RD, AD (axial diffusivity), and MD. (C) Differential impact of glioma on CST and SLF using diffusivity parameters. Only AD was significantly different between the CST and SLF, indicating that the SLF was more negatively impacted by the presence of glioma. (D) Direct point-by-point analysis along the distance of interpolated tracts estimated for the CST grouped by presence or absence of tumor to determine localized differences in diffusivity parameters. False discovery rate (FDR) was used to recalculate an adjusted critical value to control for multiple comparisons. A Dissimilarity index (DSI) was derived by calculating the ratio of significant tests. Significant points along the CST consistent were observed on the hemisphere ipsilateral to tumor in RD (*D_1_*) and FA (*D_3_*), but not AD (*D_2_*), and MD (*D_4_*). (E) Similar to *D*, using the fibers derived for the SLF. Significant points along the SLF were observed on the hemisphere ipsilateral to tumor in AD (*D_2_*) and MD (*D_4_*), but not RD (*D_1_*), and FA (*D_3_*).

Gliomas typically require resective surgical intervention [15]. Intraoperative neuronavigation is employed to improve sparing of major white matter tracts important for motor and language functions [16]. In this study, we examined two major white matter fasciculi commonly imaged in resective surgeries targeting motor areas: CST and the SLF. In Figure 2A, we analyzed glioma impact on CST white matter structure. Across the five tract parameters, tract number, RD, FA and MD were significantly affected by presence of glioma (tract number, *p* = 0.05, *t *= 2.16, df = 16; RD, *p* = 7.25e-04, *t* = 4.16, df = 16; FA, *p* = 2.25e-04,* t* = -4.73, df = 16; MD, *p* = 0.003, t = 3.39, df = 16), but AD was unchanged (*p* = 0.24, *t* = 1.21, df = 16). Glioma also induced significant axonal modifications in the SLF (Figure 2B) with statistical differences observed for tract number, RD, AD, and MD (tract number, *p* = 0.01, *t *= 2.90, df = 16; RD, *p* = 0.03,* t* = 2.44, df = 16; AD, *p* = 1.52e-04, *t* = 4.92, df = 16; MD, *p* = 0.005,* t* = 3.25, df = 16), but no change in FA (*p* = 0.78, *t* = -0.27, df = 16). In Figure 2C, we directly compared glioma impact on the CST versus the SLF and found that only AD was significantly different exhibiting a higher percent change in the SLF (*p* = 2.26e-05, *Z* = 255). Finally, to directly compare across similar normalized voxel locations within the two major white matter tracts (i.e., CST and SLF), we computed the along-tract statistics of length-normalized tracts [14]. With this analysis, we compared voxel-wise estimation of the diffusivity measures. Figure 2D_1-4_ depicts the along-tract analysis for the CST with respect to RD (green, Figure 2D_1_), AD (red, Figure 2D_2_), FA (purple, Figure 2D_3_), and MD (blue, Figure 2D_4_). Significance values were computed at each point along the tract, and false discovery rate (FDR, [13]) was used to correct the critical *p*-value for multiple comparisons. The proportion of significant points along the tract was used to generate a Dissimilarity Index $DSI = \frac{Q}{T}$ , where *Q* is total number of significant tests based on the FDR adjusted critical value and *T* is the total number of tests. For the CST, both RD and FA exhibited DIs above 0 (RD, DSI = 0.15, adjusted critical value = 0.01; FA, DSI = 0.44, adjusted critical value = 0.02). Figure 2E_1-4_, represents the along-tract analysis for the SLF. Both AD and MD exhibited high DI values (AD, DSI = 1, adjusted critical value = 0.05; MD, DSI = 0.78, adjusted critical value = 0.03). Interestingly, the along-tract indicated that the glioma-affected CST and glioma-affected SLF were differentially distinguished from their non-tumor contralateral hemisphere based on the combination of AD, RD, MD and FA.

Impact of glioma lobar localization on major white matter architecture

**Figure 3:**
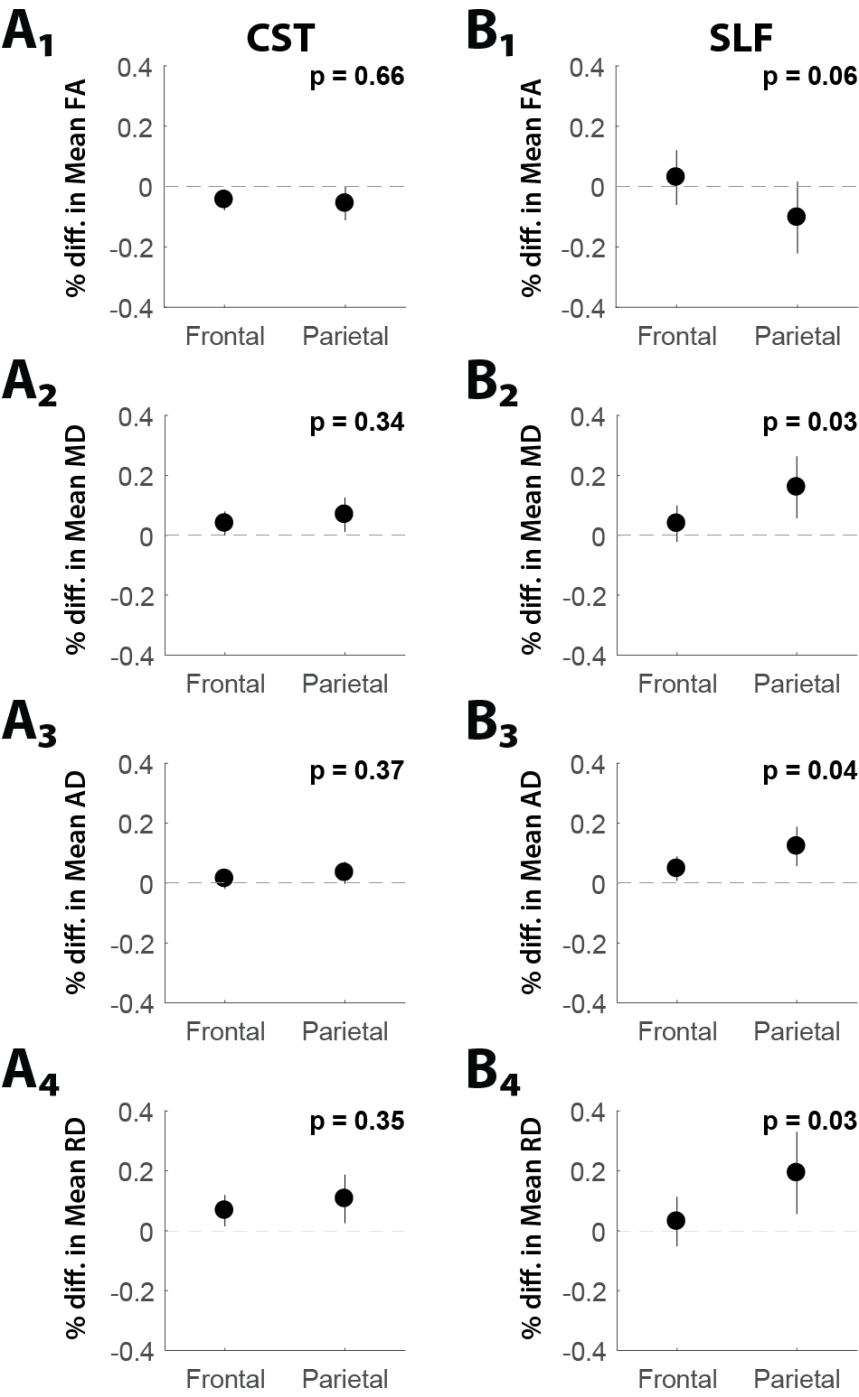
Impact of glioma lobar localization on major white matter architecture. (A_1-4_) Lobar location of glioma (i.e., frontal or parietal) did not affect the white matter parameters of the CST (corticospinal tract). (B) Lobar location of glioma (i.e., frontal or parietal) had a significantly more negative impact on some measures of SLF (superior longitudinal fasciculus) white matter architecture – MD (mean diffusivity; B*_2_*), AD (axial diffusivity; B*_3_*) and RD (radial diffusivity; B*_4_*), but not others FA (fractional anisotropy; B*_1_*), when gliomas were localized to the parietal lobe.

In Figure 3A,B, we directly compared the impact of lobar localization of glioma on degradation in the CST and SLF with respect to the frontal and parietal lobes. For all diffusivity parameters, we observed no difference on the impact on CST when comparing between gliomas localized to parietal or frontal lobes (Figure 3A_1-4_). However, for MD, AD and RD, glioma localization to the parietal lobe had greater impact on the SLF than gliomas localized to the frontal lobes (MD,* p* = 0.03, Figure 3B_2_; AD, *p* = 0.04, Figure 3B_3_; RD, *p *= 0.03, Figure 3B_4_).

## Discussion

Summary of findings

This project aimed to show the use of DTI as an anatomical/pathophysiological tool to analyze the effects of glioma. Glioma impact on the ipsilateral hemisphere showed a significant increase in RD and MD and a significant decrease in FA (Figure 1A). These suggest higher radial diffusivity and mean diffusivity and lowered magnitude of water diffusion in the tumor hemisphere or the presence of peritumoral edema. A significant increase in track number and AD was also noted on the tumor side which suggested less axonal damage to the tumor side. However, this does not match the expected outcome of this parameter, and suggests that AD may be more complex than only representing axonal integrity (variability of AD has been shown previously [17]). Similar results were found when analyzing metastasis impact. Significant increase in RD and MD and significant decrease in FA showed signs of white matter degradation on the tumor hemisphere. Significant increase in AD showed signs of less axonal damage ipsilateral to the tumor hemisphere (Figure 1C). Glioma grade III/IV impact was then compared to grade II impact on white matter integrity. No statistically significant differences were found, but a trend was seen in that grade III/IV tumors had a lower AD and FA than grade II tumors, suggesting a possible higher negative impact on white matter integrity in grade III/IV cases (Figure 1B). Comparison of glioma versus metastasis impact showed a trend of white matter degradation in both cases with regard to specific tract parameters. Glioma cases had a lower tract integrity with respect to higher mean diffusivity and lower fractional anisotropy while metastasis showed degradation with respect to higher radial diffusivity, suggesting gliomas and metastases affect white matter in unique ways that may be detectable by DTI parameters (Figure 1D). To analyze if the presence of glioma uniformly impacted major white matter pathways, glioma impact on the corticospinal tract and superior longitudinal fasciculus was studied. The differences observed between tumor impact on the SLF and CST in both scatter plot and along-tract analyses showed that the presence of glioma does not affect white matter tracts of the tumor hemisphere equally along various track parameters (Figure 2). The results showed that, in the case of the CST, lobar location did not affect the impact of glioma on the CST (Figure 3A). However, in the case of the SLF, we found that those gliomas which arise in the parietal lobe have a higher negative impact on white matter integrity of the SLF, with regards to FA, MD, and RD, than those gliomas which arise in the frontal lobe (Figure 3B).

Use of DTI in the context of gliomas – diagnosis, prognosis and aid to surgical intervention: Histopathology – has shown the degradation of neural tissue due to the presence of glioma [1]. However, a non-invasive method of determining tumor-related neural degradation would be advantageous. In the context of intracranial tumor resection, DTI has mainly been used for preoperative surgical planning [18], but increasingly is being explored for use as an anatomical/pathophysiological tool for brain tumor prognosis and analysis. However, information derived from fiber tract analysis must be interpreted with caution due to the difficulty in differentiating tumor from edema, particularly with respect to glioma. Several studies have used DTI as a diagnostic tool to classify glioma grade based on the effects these tumors have on surrounding white matter FA [19]. In addition to diagnostic analyses, the level of axonal infiltration by glioma has been studied using DTI [20]. More recent studies have expanded on this by exploring how gliomas affect white matter integrity with regard to FA, MD, and ADC (Apparent Diffusion Coefficient) [8]. These studies have helped provide insight on the various capabilities of DTI in examining tumor pathophysiology. This project further examined the information which could be extracted from DTI tumor/white matter analysis data by addressing the limitations of previous studies, which included having a larger sample size, analyzing track number, AD, and RD along with the standard DTI measures, and examining tract length anatomy with the along-tract analysis.

Implications for future studies

These results show preliminary data on the validity of the use of DTI in analyzing the impact of intracranial tumors on white matter integrity. However, a few of our observations require further investigation. Specifically, we will explore an explanation for the unexpected increase in track number and AD on the tumor side. Related to our anomalous AD finding is the more general inquiry of ascertaining the anatomical/pathological explanation for the relationship between AD and white matter integrity. Its increase likely has pathological implications, rather than only relating to axonal integrity. This study provides the groundwork for future studies focused on developing strategies to improve tumor resection using preoperative DTI to help predict or avoid functional damage from tumor resection.

Limitations

This study provided evidence for the use of DTI as an anatomical/pathophysiological tool to analyze the impact of intracranial tumor on neural milieu. However, this study had limitations which should be addressed in future studies. We saw trends when comparing glioma grade III/IV and grade II impact, but could not reach significance due to a lack of large enough sample size. Similarly, when comparing glioma and metastasis impact, a trend was seen without significance, Glioma n = 17 while metastasis n = 7. An increase in sample size for metastasis cases could potentially show a significant difference between glioma and metastasis pathophysiology on white matter integrity. The main limitation of this project was the absence of histological confirmation of tumor impact on white matter integrity for each case. This would have helped confirm the results of the DTI analysis by giving physical observable evidence of white matter degradation.

## Conclusions

In this study, we observed that DTI tractography analysis showed signs of global and focal white matter degradation on the ipsilateral tumor hemisphere. Results also showed increased white matter degradation of the SLF in cases in which gliomas arise in the parietal lobe. These results further validate DTI’s use as a noninvasive anatomical/pathophysiological tool to analyze intracranial tumor impact on neural tissue. However, histological confirmation of white matter degradation in the sample and an increased sample size would be ideal to strengthen this study.

## References

[1] Louis DN, Perry A, Reifenberger G (2016). The 2016 World Health Organization Classification of Tumors of the Central Nervous System: a summary. Acta Neuropathol.

[2] Deighton RF, McGregor R, Kemp J (2010). Glioma pathophysiology: insights emerging from proteomics. Brain Pathol.

[3] Raza SM, Lang FF, Aggarwal BB (2002). Necrosis and glioblastoma: a friend or a foe? A review and a hypothesis. Neurosurgery.

[4] Schneider SW, Ludwig T, Tatenhorst L (2004). Glioblastoma cells release factors that disrupt blood-brain barrier features. Acta Neuropathol.

[5] Essayed WI, Zhang F, Unadkat P (2017). White matter tractography for neurosurgical planning: a topography-based review of the current state of the art. NeuroImage Clin.

[6] Bammer R (2003). Basic principles of diffusion-weighted imaging. Eur J Radiol.

[7] Alexander AL, Lee JE, Lazar M (2007). Diffusion tensor imaging of the brain. Neurotherapeutics.

[8] Mori S, Frederiksen K, van Zijl PCM (2002). Brain white matter anatomy of tumor patients evaluated with diffusion tensor imaging. Ann Neurol.

[9] Yeh F-C, Tseng W-YI (2013). Sparse solution of fiber orientation distribution function by diffusion decomposition. PLoS One.

[10] Jiang H, van Zijl PCM, Kim J (2006). DtiStudio: resource program for diffusion tensor computation and fiber bundle tracking. Comput Methods Programs Biomed.

[11] Tzourio-Mazoyer N, Landeau B, Papathanassiou D (2002). Automated anatomical labeling of activations in SPM using a macroscopic anatomical parcellation of the MNI MRI single-subject brain. Neuroimage.

[12] Wakana S, Caprihan A, Panzenboeck MM (2007). Reproducibility of quantitative tractography methods applied to cerebral white matter. Neuroimage.

[13] Benjamini Y, Hochberg Y (1995). Controlling the false discovery rate: a practical and powerful approach to multiple testing. J R Statist Soc.

[14] Colby JB, Soderberg L, Lebel C (2012). Along-tract statistics allow for enhanced tractography analysis. Neuroimage.

[15] Hottinger AF, Stupp R, Homicsko K (2014). Standards of care and novel approaches in the management of glioblastoma multiforme. Chin J Cancer.

[16] Raffa G, Conti A, Scibilia A (2017). Functional reconstruction of motor and language pathways based on navigated transcranial magnetic stimulation and DTI fiber tracking for the preoperative planning of low grade glioma surgery: a new tool for preservation and restoration of eloquent networks. Trends in Reconstructive Neurosurgery. Acta Neurochirurgica Supplement.

[17] Racine AM, Adluru N, Alexander AL (2014). Associations between white matter microstructure and amyloid burden in preclinical Alzheimer’s disease: a multimodal imaging investigation. NeuroImage Clin.

[18] Sollmann N, Wildschuetz N, Kelm A (2017). Associations between clinical outcome and navigated transcranial magnetic stimulation characteristics in patients with motor-eloquent brain lesions: a combined navigated transcranial magnetic stimulation–diffusion tensor imaging fiber tracking approach. J Neurosurg.

[19] Davanian F, Faeghi F, Shahzadi S (2017). Diffusion tensor imaging for glioma grading analysis of fiber density index. Basic Clin Neurosci J.

[20] Price SJ, Burnet NG, Donovan T (2003). Diffusion tensor imaging of brain tumors at 3T: a potential tool for assessing white matter tract invasion?. Clin Radiol.

